# Anthropometrics Analysis of Mental Foramen and Accessory Mental Foramen in Zambian Adult Human Mandibles

**DOI:** 10.1155/2019/9093474

**Published:** 2019-07-16

**Authors:** Balakrishnan Subramanian, Severine N. Anthony, Lumamba Mubbunu, Chitinti Hachombwa, Majuto S. Mlawa, Mudhihiri M. Majambo, Rajab M. Sasi

**Affiliations:** ^1^Department of Basic Medical Sciences, Michael Chilufya Sata School of Medicine, Copperbelt University, P.O. Box 71191, Ndola, Zambia; ^2^Department of Dental Clinical Sciences, Michael Chilufya Sata School of Medicine, Copperbelt University, P.O. Box 71191, Ndola, Zambia

## Abstract

The mental foramen (MF) and accessory mental foramen (AMF) are the strategically important landmarks during surgical interventions and anaesthetic nerve blocks procedures involving the mental nerve. The study aimed at evaluating anthropometrics of MF and AMF in Zambian adult human mandibles and it was cleared for ethics from TDRC Ethics Review Committee (Reg. No.: 00002911; FWA: 00003729). A total of 33 Zambian adult human mandibles were evaluated for shape, position, and direction of opening of foramen. All measurements were performed using a Digital Vernier Calliper and statistically analysed for per cent frequency and mean and standard deviations, and we performed the one sample* t*-test for comparative analysis. Data were considered significant at p<0.05. All mandibles that were examined had bilateral MF while unilateral AMF was found in two mandibles (6%). The foramens were mostly oval in shape and their most common position was between the second premolar and first molar and the most common orientation was posterior-superior. The comparative analysis of mandibular anthropometrics showed significant variations (p<0.05) with different ethnic groups. The findings emphasize the ethnic variations and edify that the foramen position is not always as stated in reference textbooks. The clinical creditability of the study is cautioning the surgeons on possible variations of the MF and AMF anthropometrics compared to existing literature in order to avoid any unforeseen injury related to anaesthesia or dental surgeries. Further studies with large sample sizes representing whole country are recommended to establish the standard MF and AMF anthropometrics of Zambian population.

## 1. Introduction

The mental foramen (MF) is an oval or circular opening on the body of the mandible where the mandibular canal terminates. It is an exit for the mental nerve and blood vessels, which are terminal branches of inferior alveolar nerve, artery, and vein. The mental nerve provides innervations of the lower teeth, lip, gingival, and lower face [1, 2]. The MF is an important anatomical landmark during osteotomy procedures, anesthetic nerve blocks, and prevention of neurovascular complications after invasive procedures on the lower jaw. Its anatomy is also useful in evaluating the morphometric symmetry of the mental triangle, microscopic and macroscopic morphology, bone remodelling activity, and paleoanthropologic features of the facial skeleton in different populations [3].

The MF is usually located in the body of the mandible at an equal distance from the superior and inferior border below or between the apex of the first and second premolar [2, 4, 5]. The direction of opening of the foramen from the inferior alveolar has been shown to be pointing posteriorly outward and upward [4, 6]. Variability in the location of MF has been documented in different literature with the tendency of being more posterior in blacks than in whites and between the second premolar and first molar [7]. A study on Tanzanian population revealed that the most frequent locations for MF were below the apex of the second premolar and between the 2^nd^ premolar and 1^st^ molar. The MF was asymmetrically located between the right and left sides and predominantly oval. The direction of opening was mostly superior and posterosuperior and rarely labial, mesial, or posterior [4]. Another study on Zimbabwean population found that the MF was mostly oval shaped and the frequency of occurrence was highest below the lower 2^nd^ premolar on the right side and between 2^nd^ premolar and 1^st^ molar on the left side [8]. In a study done on Malawian population, the MF was found to be oval in shape, oriented posterosuperiorly, and located inferior to the 2^nd^ premolar tooth and bilaterally symmetrical in a majority of cases. Its vertical position was slightly below the midpoint of the distance between the lower border of the mandible and the alveolar margin [9].

Any foramen in addition to MF in the body of mandible is known as accessory mental foramen (AMF) and it tends to exist in the apical area of the first molar and posterior or inferior area of the mental foramen. As AMF is due to branching of mental nerve before passing through MF, its shape, size, and verification of its existence would prevent accessory nerve injury during periapical surgery [2, 10]. The potential severe complication of injury of the accessory mental foramen (AMF) is sensory disturbance of the lower lip [11]. Studies [12, 13] have reported AMF incidence to range from 1.4% to 9.7% with an exception of one on Japanese population, which reported very high incidence of 12.5% [14]. The distances between MF and AMF were reported to range between 0.67mm and 5.74mm [14]. Ethnic variations in relation to AMF have also been reported [12]. Absent AMF are a more common variation than MF absence in humans and the frequent reasons for absence may range from atrophy, posttraumatic fibrosis, osteoblastic hyperplasia, geriatric bony resorption, or congenital agenesis [3].

Hence location, size, shape, position, and incidence of MF and AMF would facilitate the dental surgeon to apply nerve block in different surgical procedures involving lower jaw. The anatomical research reports on variations in anthropometrics of MF and AMF between race and geographical location signifying the need to establish local values. Currently there are no established values on MF and AMF for Zambian population. Therefore, this study is aimed at evaluating anthropometrics of MF and AMF in Zambian adult human mandibles to establish specific MF and AMF anthropometrics of Zambian population in order to make a gateway to add them to the medical literature.

## 2. Materials and Methods

### 2.1. Study Samples

A total of 33 Zambian adult cadavers out of 35 availed in the Anatomy Laboratory of Michael Chilufya Sata School of Medicine (MCS SoM), which met the inclusion criteria, that is, dentate adult mandibles, were considered for the study. The cadavers used in the study were legally permitted for the use of education and research purposed at the MCS SoM and the appropriate permission was obtained from the Dean, MCS SoM for their use in the study.

### 2.2. Ethical Compliance

Ethical clearance for this anatomical study (Approval Reference No. TRC/C4/04/2017) was sought from TDRC Ethics Review Committee (Reg. No.: 00002911; FWA: 00003729), Ndola, Zambia. Handling of cadavers was done only in Anatomy laboratory dissecting hall and no body parts were moved out of the dissecting hall. The cadaver was treated with respect as to the respect given to human life. The information about the cadaver in terms of names or names of the relatives was not sought or collected and only information about age, sex, and race was used for the study.

### 2.3. Preparation of Mandibles

The mandibles were disarticulated, cleaned, and then rinsed in 70% ethanol solution and allowed to dry for 24hrs. The mandibles were chosen according to the following criteria:

1. As a minimum, all mandibular teeth from right first molar to left first molar were present
2. All mandibular teeth from right first molar to left first molar were in a reasonably normal position and alignment

### 2.4. Anthropometric Measurements

The following observations and measurements were performed directly on each dry mandible for both left and right sides. The measurements were made using Digital Vernier Calliper, which was calibrated before every measurement.

#### 2.4.1. Number Present

Numbers of MF and AMF on each dry mandible for both left and right sides were visually scored and recorded.

#### 2.4.2. Size

The greatest horizontal and vertical dimensions of each MF and AMF were recorded.

#### 2.4.3. Orientation

The direction of opening of each mental canal through the lateral surface of the mandible was visually determined and recorded.

#### 2.4.4. Position

**Figure 1:**
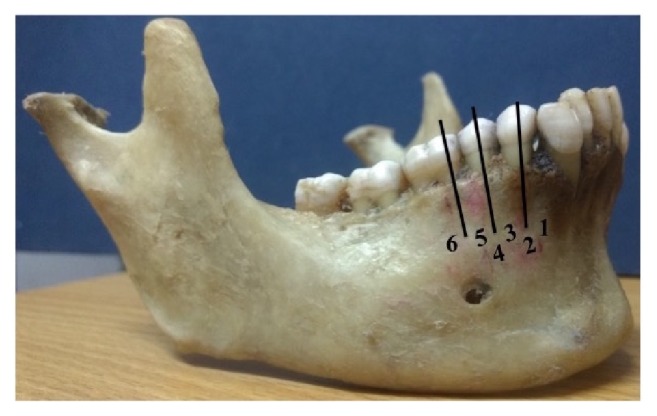
The position of the mental foramen with respect to the teeth of the lower jaw. Position 1: situated anterior to the first premolar; position 2: in line with the first premolar; position 3: between the first and second premolar; position 4: in line with second premolar; position 5: between the second premolar and first molar; position 6: in line with the first molar.

The positions of the MF and AMF in relation to the mandibular teeth of the lower jaw were recorded as follows (Figure 1):

  Position 1: situated anterior to the first premolar
  Position 2: in line with the first premolar
  Position 3: between the first and second premolar
  Position 4: in line with second premolar
  Position 5: between the second premolar and first molar
  Position 6: in line with the first molar

#### 2.4.5. Mandibular Anthropometrics from Defined Landmarks

**Figure 2:**
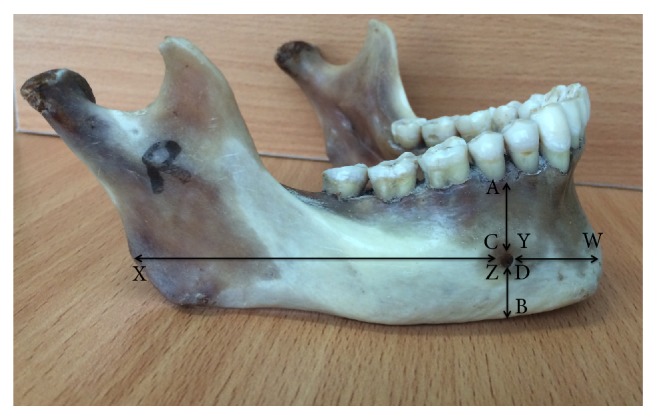
Mandibular anthropometrics from defined landmarks. AB: distance from alveolar crest to lower border of mandible, AC: distance from alveolar crest to upper margin of mental foramen (MF), BD: distance from lower border of mandible to lower margin of MF, WX: distance from symphysis menti to posterior border of ramus of mandible, WY: distance from symphysis menti to medial margin of MF (anterior chord), and XZ: distance from posterior border of ramus of mandible to lateral margin of MF (posterior chord).

The positions of MF and AMF were evaluated by measuring the distance of MF/AMF (in mm) from various landmarks including symphysis menti, alveolar crest, posterior border of the ramus of mandible, and lower border of mandible (Figure 2).

The distance measurements included the following:

  AB: distance from alveolar crest to lower border of mandible
  AC: distance from alveolar crest to upper margin of MF/AMF
  BD: distance from lower border of mandible to lower margin of MF/AMF
  WX: distance from symphysis menti to posterior border of ramus of mandible
  WY: distance from symphysis menti to medial margin of MF/AMF (anterior chord)
  XZ: distance from posterior border of ramus of mandible to lateral margin of MF/AMF (posterior chord)
  VD: vertical diameter of the MF/AMF = AB – (AC+BD)
  HD: horizontal diameter of the MF/AMF = WX – (XZ+WY)

The relative position of MF/AMF was found by subtracting the average distance from symphysis menti to medial margin of MF/AMF (WY) with length of the mandibles, i.e., ratio of WY/WX.

#### 2.4.6. Study of Ethnic Variations

The positions of MF and AMF in relation to the mandibular teeth and mandibular anthropometrics from defined positions were compared with reported values of different ethnic groups on different geographic locations, viz., Malawians [9], Turkish [15], Brazilians [16], Thais [17], Indians [10, 18], Sri Lankans [19, 20], Chines [21], and Europeans [21].

### 2.5. Statistical Analysis

Statistical analysis was done using IBM-SPSS software version 20. The most prevalent shape, position, and orientations were found by simple frequency analysis. The MF and AMF diameters for size and distances from defined positions were measured using a Digital Vernier Calliper (in mm) and statistically analysed for per cent frequency and mean and standard deviations (SD). The comparative analyses of mandibular anthropometrics from defined positions were performed using a one-sample* t*-test. Data were considered statistically significant at p<0.05.

## 3. Results

**Figure 3:**
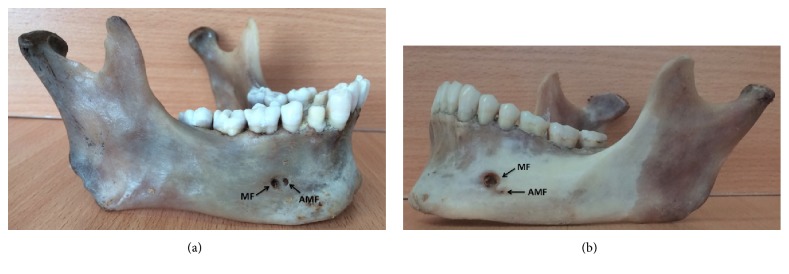
Adult Zambian human mandibles found with MF and AMF. (a) Mandibles found with MF and AMF on the right side. (b) Mandibles found with MF and AMF on the left side.

A total of 33 Zambian adult human mandibles of known sex that included 31 male mandibles and 2 female mandibles were evaluated for MF and AMF anthropometrics measurements. Since the proportion of female to male samples was too low (6%), the data were not analysed and stratified by sex. The frequency distribution of MF and AMF and their shapes in Zambian adult human mandibles are shown in Table 1. The study found one mandible (n=33, 3%) with AMF on the right side (Figure 3(a)) and another (n=33, 3%) with AMF on the left side (Figure 3(b)) along with bilateral MF, and the rest were only with bilateral MF. The AMF/MF ratio was 3% (n=33) on both sides of mandibles. The shapes of the MF and AMF were mainly oval on both sides of mandibles.

The dimensions of the MF and AMF (Table 2) on the mandibles were determined by measuring their horizontal diameters (HD) and vertical diameters (VD). The average HD of the MF was 3.6±0.9mm on the right side and 3.8±0.9mm on the left side. The average VD of the MF was 2.8±0.7mm on the right side and 3.2±1.1mm on the left side. The HD and VD of AMF found on the left side were 1.9mm and 0.8mm, respectively, while for another one found on the right side they were 1.3mm and 2.1mm, respectively.

The study found that the most common position (Table 3) for MF on the left side was position 5 (f=17, 51.5%) followed by position 4 (f=11, 33.3%) and the right side was position 5 (f=15, 45.5%) followed by position 4 (f=8, 24.2%). No MF was noted in positions 1 and 2. The MF was symmetrical in 23 (n=33, 69.7%) mandibles with the remaining 10 (n=33, 30.3%) being asymmetrical. For the symmetrically placed MF, the most common position was position 5 (f=13, 39.4%), followed by position 4 (f=6, 18.2%). The AMF found on the left side was at position 5 (f=1, 3%) and the right side at position 5 (f=1, 3%).

**Figure 4:**
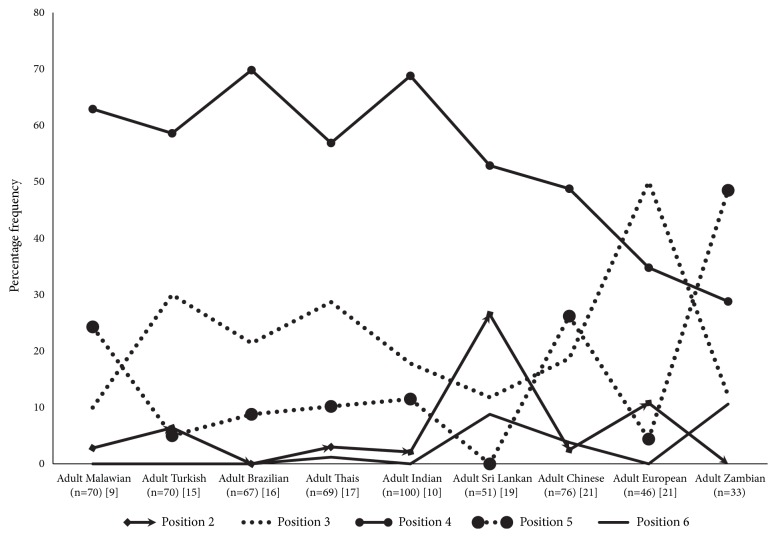
The position of MF in relation to mandibular teeth among different ethnic groups on different geographic locations: comparative analysis (frequency in percentage) with other reported values. Position 2: in line with the first premolar, position 3: between the first and second premolar, position 4: in line with second premolar, position 5: between the second premolar and first molar, and position 6: in line with the first molar.

The positions of MF in relation to mandibular teeth among different ethnic groups on different geographic locations were compared using per cent frequency (Figure 4) and it was found that the majority of groups had their mental foramen in line with second premolar (position 4) and between first and second premolar (position 3). While position 4 was the most common position of the mental foramen, position 3 was the next common and vice versa. However the most common position found in the present study was position 5 and the next common position was position 4.

The frequency distributions of MF and AMF directions of opening (orientation) are shown in Table 4. The most common direction of opening of the MF on the right side was posterior-superior (f=16, 48.5%), followed by labial (f=9, 27.2%), and for the left side it was posterior-superior (f=14, 42.4%), followed by labial (f=12, 36.4%). The direction of opening of AMF on the left side was posterior (f=1, 3%) while that on the right side was posterior-superior (f=1, 3%).

**Figure 5:**
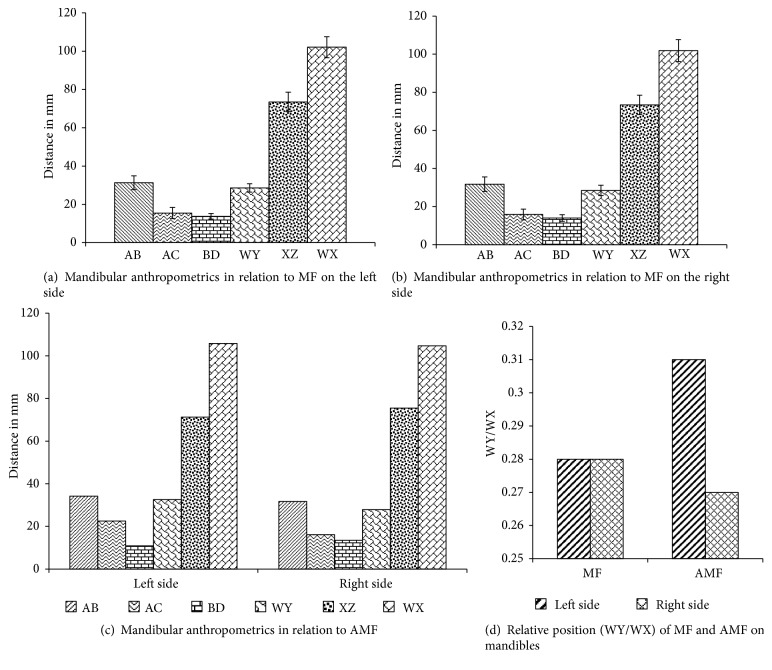
Mandibular anthropometric measurements on Zambian adult human mandibles (n=33). AB: alveolar crest, lower border of mandible; AC: alveolar crest, upper margin of foramen; BD: lower border mandible, lower margin foramen; WY: symphysis menti, medial margin of foramen; XZ: posterior border ramus, lateral margin foramen; WX: length of mandibles; WY/WX: relative position. The error bars represent the standard deviation of measurements.

The mandibular anthropometric measurements (the distance of MF and AMF from defined landmarks) on Zambian adult human mandibles (n=33) are presented in Figure 5. The MF were positioned at mean AC of 15.5±2.9mm and mean BD of 13.7±1.5mm, likewise the AMF at AC and BD of 16.1mm and 13.5mm, respectively, on the left side. The mean AC and BD were 15.9±2.9mm and 13.9±1.7mm, respectively, for MF and 16.1mm and 13.5mm, respectively, for AMF on the right side. The mean WY was 28.6±2.2mm for MF and 32.6mm for AMF on the left side and 28.5±2.7mm for MF and 27.9mm for AMF on the right side. The mean XZ on the left side was 73.5±5.1mm for MF and 71.3mm for AMF and the right side 73.4±5.1mm for MF and 75.5mm for AMF. The average lengths of mandible were 102.1±5.5mm on the left side and 101.9±5.8mm on the right side. The relative position of MF on both left and right side was 0.28±0.02 but it was 0.31 on the left side and 0.27 on the right side for AMF.

**Figure 6:**
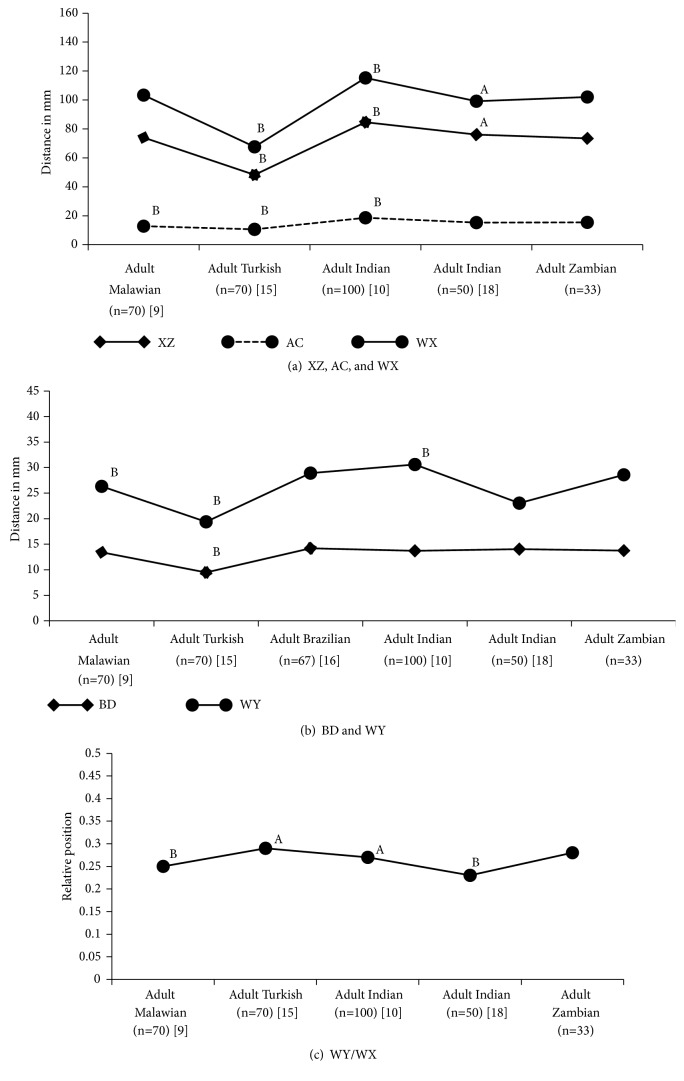
The mandibular anthropometrics of the left side of mandibles among different ethnic groups on different geographic locations: comparative analysis with other reported values. AC: distance (in mm) from alveolar crest to upper margin of mental foramen, BD: distance (in mm) from lower border of mandible to lower margin of mental foramen, WY: distance (in mm) from symphysis menti to medial margin of mental foramen, XZ: distance (in mm) from posterior border of ramus of mandible to lateral margin of mental foramen, WX: length of mandibles, and WY/WX: relative position. ^A^Significant at p<0.05; ^B^significant at p<0.001.

**Figure 7:**
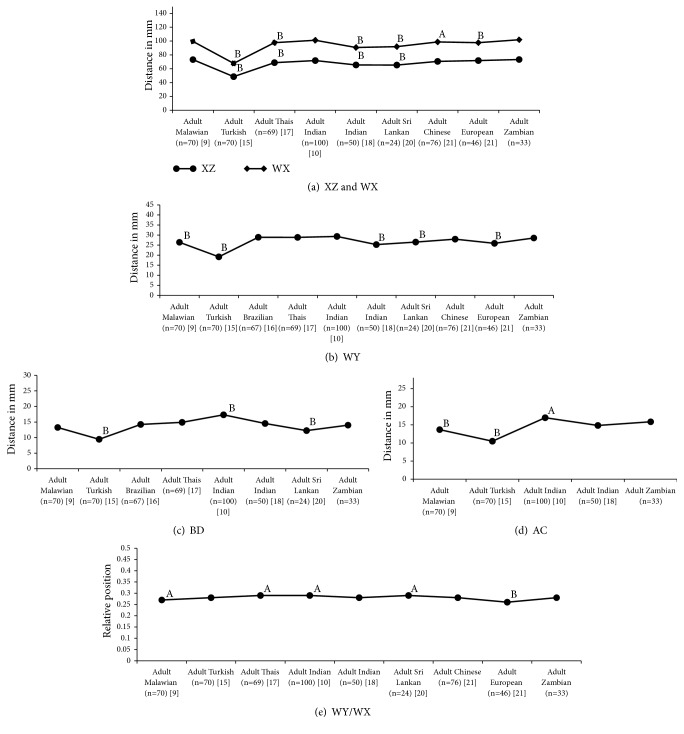
The mandibular anthropometrics of the right side of mandibles among different ethnic groups on different geographic locations: comparative analysis with other reported values. AC: distance (in mm) from alveolar crest to upper margin of mental foramen, BD: distance (in mm) from lower border of mandible to lower margin of mental foramen, WY: distance (in mm) from symphysis menti to medial margin of mental foramen, XZ: distance (in mm) from posterior border of ramus of mandible to lateral margin of mental foramen, WX: length of mandibles, and WY/WX: relative position. ^A^Significant at p<0.05; ^B^significant at p<0.001.

The one sample* t*-test comparative analysis (Figures 6 and 7) of mean distances of MF from defined landmarks, length of mandibles, and relative position of MF on mandibles with other reported values of different ethnic groups on different geographic locations, viz., Malawi, Turkey, Brazil, Thailand, Srilanka, China, Europe, and India, revealed that the study findings are significantly varying from the reported values of different ethnic groups on different geographic locations. The average length of mandibles was ranging from 67.64mm to 115.3mm on the left side and 67.76mm to 101.89mm on the right side with lowest on Turkish and highest on Indians. The relative position of the MF was ranging from 0.26 to 0.29 on the right side of mandibles and from 0.23 to 0.29 on the left side of mandibles.

## 4. Discussion

The precise identification of the mental foramen and its anthropometric characteristics are important in both diagnostic and clinical procedures of the mandible. The standard anatomy texts state that the mental foramen is most commonly found between the apices of the first and second lower premolar [2]. Although this is in accord with some European populations, it does not take into account of a large body of evidence with reference to other populations [9, 10, 15, 17, 19]. Furthermore, racial variation in the most common position of the mental foramen was clearly demonstrated by comparative studies among Chinese, European, and Indian population [21] and there was an interesting finding that the mental foramen was positioned more posteriorly in blacks than in whites [7, 22]. In the present study, we have evaluated the anthropometrics of MF and AMF in Zambian adult human mandibles to establish specific MF and AMF anthropometrics of Zambian population.

The study found the presence of unilateral AMF along with bilateral MF in two cases (6%, n=33) and the rest were presented with bilateral MF. There was no case with absence of foramen and there were no differences in the prevalence of MF and AMF between left and right sides of mandibles. Though our findings are in accord with most of the earlier reports [10, 12, 23], it disagrees with the study that reported the absence of MF [3, 22]. It is also important to note that the AMF/MF ratio was 3% (n=33) on both sides of mandibles. According to Iwanaga* et al*. [11], the AMF/MF ratio and positional relations of AMF to the MF could help in assessing the risk of neurosensory disturbance of lower lip. The shape of MF was seen to be predominantly oval in Tanzanian [4], Zimbabwean [8], and Malawian [9] adult population. Similar result was observed in the present study where the shape of the MF on both sides of the jaw was mainly oval (80% in the left side and 73.3% in the right side) and the AMF found on both the sides were oval in shape. In contrast, the studies with Indian adults [10, 18] revealed that the MF was predominantly more circular (round) in shape.

The study also found that the overall mean dimension of MF was 3.35mm, while it was 3.18mm and 3.79mm in the Zimbabwean [8] and Malawian [9] adult populations, respectively. The observed average HD of the MF was 3.7mm, while it was ranging from 3.26mm to 3.41mm in the Asian populations [19, 24, 25]. It is also important to note that a wider HD of 5.16mm [26] and a lower HD of 2.69mm [10] were also reported. The average VD of the MF observed in the study was 3mm, while it was ranging from 2.13mm to 2.61mm in the Asian populations [19, 24–26]. The mean dimension of AMF found in the study was 1.5mm, while it was 0.5 - 1mm in Indian adult population [10, 23].

The position of the MF varies considerably by geographic location and nationality [9, 10, 15, 17, 19]. The knowledge on the position of MF to specific ethnic group is very important especially during implantology so that one can avoid putting implant in the MF. The most common position of MF was position 5 that is between the second premolar and first molar and the next common position was position 4 that is in line with second premolar, while the position of AMF on both cases were position 5. Positions 1 and 2 were not observed in this study and this is in line with the report that the position of the MF was more posterior in blacks than whites [22]. The comparative study of position of MF in relation to mandibular teeth with selected reports of different ethnic groups revealed significant differences among ethnic groups on different geographic locations [9, 10, 15–17, 19, 21]. Therefore, the variability of the position of the MF should be considered when undertaking periodontal or endodontic surgery in the area from the canine to the mesial root of the first molar. Though caution has to be taken, it does not exclude the need of radiographs prior to any periodontal or endodontic surgery.

Previous studies on mandibles of different ethnic groups [4, 9, 16, 26] revealed that the direction of opening (orientation) of the MF was mainly posterior-superior. Similar result was observed in the present study where the most common direction of opening (orientation) of MF was posterior-superior (46%) and the next common was labial (31.8%), while it was posterior and posterior-superior, respectively, for the AMF found on the left and right sides of mandibles.

According to the anthropometric measurements in the study, the MF was found to be at an equal distance on both sides of the mandible but it was not true in case of AMF. The mean anterior chord (WY) and posterior chord (XZ) obtained were 28.5mm and 73.4mm, respectively, for MF on both sides of mandibles, while they were 32.6mm and 71.3mm on the left side and 27.9mm and 75.5mm on the right side for AMF. The mean length of mandibles was 101.9mm. It was also noted that the mean position of MF was found 13.86mm above the lower border of the mandible and 15.66mm below the alveolar ridge on both sides of mandibles but the AMF was found 10.9mm and 13.5mm above the lower border of the mandible and 22.5mm and 16.1mm below the alveolar ridge, respectively, on the left and right sides. The relative position of the MF and AMF in the mandible was obtained by calculating the ratio of WY/WX and it was 0.28 for MF on both sides of the mandible but it was 0.31 on the left side and 0.27 on the right side for AMF. Previous studies [9, 18] reported that the MF lies approximately below one-fourth of the distance from the symphysis menti to the posterior border of the mandible on the left side, which is slightly smaller than the calculated value of the present study.

A comparative study on mandibular anthropometrics of selected reports of different ethnic groups [9, 10, 15–18, 20, 21] revealed significant variation among different ethnic groups on different geographic locations. In most cases, the mean distances of the MF from defined landmarks on the mandibles, length of mandibles, and relative position of MF on mandibles were significantly differed among different ethnic groups from different geographic locations. The relative position of the MF was ranging from 0.26 to 0.29 on the right side of mandibles and from 0.23 to 0.29 on the left side of mandibles. It was slightly higher on the right of mandibles and slightly lower on the left of mandibles for adult Asians while it was slightly lower on the right of mandibles for adult Europeans when compared to adult Africans. Though the lengths of mandibles on right side are similar among Malawians (also on left side), Zambians, and Indians, the MF lies further forward in adult Malawians and backward in adult Indians when compared to adult Zambians. In contrast, the lengths of mandibles on right side are significantly different among Turkish, Chinese, and Zambians but the relative position of the MF was in agreement. Hence, this study evidences for wide variation of MF and AMF anthropometrics of adult Zambians compared to other geographic locations and ethnicities.

## 5. Conclusion

As a preliminary attempt, the study has established specific MF and AMF anthropometrics for adult humans in Copperbelt province of Zambia. There are variations in the MF characteristics of studied Zambian mandibles compared to established values worldwide and within the region. The results of the present study highlight the racial differences in the most common position and relative position of the MF observed among different populations in the different geographic locations. The clinical creditability of the study is cautioning the surgeons on possible variations of the MF and AMF anthropometrics compared to existing literatures in order to avoid any unforeseen injury related to anaesthesia or dental surgeries. Although the study has the limitations that the findings are based on a small sample size, which is also having too low female to male proportion which was due to challenges in acquiring cadavers, the data was normally distributed and showed normal variations so the information on anthropometrics of MF and AMF presented in this article can help anatomists, prosthodontists, orthodontists, surgeons, forensic odontologists, and paleoanthropologists to predict the position of the MF and AMF and perform both diagnostics and safer clinical procedures led on the mandibles of Zambian population. However, it is recommended for further studies with large sample sizes representing whole country to establish the standard MF and AMF anthropometrics of Zambians.

## Figures and Tables

**Table 1:** Frequency distribution of number and shapes of MF and AMF and AMF/MF ratio in Zambian adult human mandibles (n=33).

Variable		Left side		Right side	
		F	%	F	%
Number	MF alone	32	97.0	32	97.0
	MF +AMF	1	3.0	1	3.0
	AMF/MF ratio	0.03	3.03	0.03	3.03

Shape of MF	Round	8	24.2	9	27.3
	Oval	25	75.8	24	72.7

Shape of AMF	Oval	1	3%	1	3%

MF, Mental foramen; AMF, Accessory mental foramen; F, Frequency; %, Percentage

**Table 2:** Dimensions of the MF and AMF in Zambian adult human mandibles (n=33).

Dimensions (mm)		Left side		Right side		Both	
		HD	VD	HD	VD	HD	VD
MF	Minimum	2.2	1.5	1.6	1.6	1.6	1.5
	Maximum	5.9	5.6	5.2	4.0	5.9	5.6
	Average	3.8	3.2	3.6	2.8	3.7	3.0

AMF		1.9	0.8	1.3	2.1	-	-

MF, Mental foramen; AMF, Accessory mental foramen; HD: Horizontal diameter, VD: Vertical diameter

**Table 3:** Frequency of MF and AMF positions in relation to mandibular teeth in Zambian adult human mandibles (n=33).

Position		Left side		Right side	
		F	%	F	%
MF	3	2	6.1	6	18.2
	4	11	33.3	8	24.2
	5	17	51.5	15	45.5
	6	3	9.1	4	12.1

AMF	5	1	3	1	3

MF: mental foramen, AMF: accessory mental foramen, F: frequency, and %: percentage. Position 1: situated anterior to the first premolar. Position 2: in line with the first premolar. Position 3: between the first and second premolar. Position 4: in line with second premolar. Position 5: between the second premolar and first molar. Position 6: in line with the first molar. No MF was found in position 1 and 2.

**Table 4:** Frequency distribution of MF and AMF directions of opening in Zambian adult human mandibles (n=33).

Direction of opening		Left side		Right side	
		F	%	F	%
MF	Superior	4	12.1	3	9.1
	Posterior	1	3	0	0
	Labial	12	36.4	9	27.2
	Mesial	0	0	1	3
	Posterior-superior	14	42.4	16	48.5
	Anterior-superior	2	6.1	2	6.1
	Posterior-inferior	0	0	2	6.1

AMF	Posterior	1	3	0	0
	Posterior-superior	0	0	1	3

MF: Mental foramen, AMF: Accessory mental foramen, F: Frequency, %: Percentage

## Data Availability

The data used to support the findings of this study are included within the article.

## Abbreviations

MF: Mental foramen
AMF: Accessory mental foramen
SD: Standard deviation.

## References

[1] Bruce Bavitz J., Harn S. D., Hansen C. A., Lang M. (1993). An anatomical study of mental neurovascular bundle-implant relationships. *The International Journal of Oral & Maxillofacial Implants*.

[2] Standring S. (2015). *Gray's Anatomy International Edition: The Anatomical Basis of Clinical Practice*.

[3] Hasan T., Fauzi M., Hasan D. (2010). Bilateral absence of mental foramen—a rare variation. *International Journal of Anatomical Variations*.

[4] Fabian F. M. (2007). Position, shape and direction of opening of the mental foramen in dry mandibles of Tanzanian adult black males. *Italian Journal of Anatomy and Embryology*.

[5] Junior E. M. O., Araújo A. L., Da Silva C. M., Sousa-Rodrigues C. F., Lima F. J. (2009). Morphological and morphometric study of the mental foramen on the M-CP-18 Jiachenjiang point. *International Journal of Morphology*.

[6] Haghanifar S., Rokouei M. (2009). Radiographic evaluation of the mental foramen in a selected Iranian population. *Indian Journal of Dental Research*.

[7] Laher A. E., Motara F., Moolla M. (2016). The ultrasonographic determination of the position of the mental foramen and its relation to the mandibular premolar teeth. *Journal of Clinical and Diagnostic Research*.

[8] Mbajiorgu E. F., Mawera G., Asala S. A., Zivanovic S. (1998). Position of the mental foramen in adult Black Zimbabwean mandibles: A clinical anatomical study. *Central African Journal of Medicine*.

[9] Igbigbi P., Lebona S. (2005). The position and dimensions of the mental foramen in adult Malawian mandibles. *West African Journal of Medicine*.

[10] Singh R., Srivastav A. K. (2010). Study of position, shape, size and incidence of mental foramen and accessory mental foramen in Indian adult human skulls. *International Journal of Morphology*.

[11] Iwanaga J., Kikuta S., Tanaka T., Kamura Y., Tubbs R. S. (2019). Review of risk assessment of major anatomical variations in clinical dentistry, Part I: accessory foramina of the mandible. *Clinical Anatomy*.

[12] Sawyer D. R., Kiely M. L., Pyle M. A. (1998). The frequency of accessory mental foramina in four ethnic groups. *Archives of Oral Biolog*.

[13] Paraskevas G., Mavrodi A., Natsis K. (2015). Accessory mental foramen: an anatomical study on dry mandibles and review of the literature. *Journal of Oral and Maxillofacial Surgery*.

[14] Toh H., Kodama J., Yanagisako M., Ohmori T. (1992). Anatomical study of the accessory mental foramen and the distribution of its nerve. *Okajimas Folia Anatomica Japonica*.

[15] Yesilyurt H., Aydinilioglu A., Kavakli A. (2008). Local differences in the position of the mental foramen. *Folia Morphologica*.

[16] Amorim M. M., Prado F. B., Borini C. B. (2008). The mental foramen in dentate and edentulous Brazilians mandible. *International Journal of Morphology*.

[17] Apinhasmit W., Methathrathip D., Chompoopong S., Sangvichien S. (2006). Mental foramen in Thais: an anatomical variation related to gender and side. *Surgical and Radiologic Anatomy*.

[18] Mishra A. B., Mittal L. (2015). Anthropometry study on mental foramen in human mandible. *International Journal of Science and Research (IJSR)*.

[19] Ilayperuma I., Nanayakkara G., Palahepitiya N. (2009). Morphometric analysis of the mental foramen in adult Sri Lankan mandibles. *International Journal of Morphology*.

[20] Prabodha L. B. L., Nanayakkara B. G. (2006). The position, dimensions and morphological variations of mental foramen in mandibles. *Galle Medical Journal*.

[21] Santini A., Alayan I. (2012). A comparative anthropometric study of the position of the mental foramen in three populations. *British Dental Journal*.

[22] Cutright B., Quillopa N., Schubert W. (2003). An anthropometric analysis of the key foramina for maxillofacial surgery. *Journal of Oral and Maxillofacial Surgery*.

[23] Rajkohila J., Daniel P., Ambikaipakan S., Rabi S. (2018). Morphological and morphometric analysis of accessory mental foramen in dry human mandibles of south indian population. *Indian Journal of Dental Research*.

[24] Agarwal D. R., Gupta S. B. (2011). Morphometric analysis of mental foramen in human mandibles of South Gujarat. *People's Journal Of Scientific Research*.

[25] Kadel M., Sedhain B. P., Dangol P. M. (2016). Morphometric analysis of mental foramen in human dry mandibles of nepalese population. *Asian Journal of Medical Sciences*.

[26] Budhiraja V., Rastogi R., Lalwani R., Goel P., Bose S. C. (2013). Study of position, shape, and size of mental foramen utilizing various parameters in dry adult human mandibles from north. *ISRN Anatomy*.

